# Melatonin application alleviates adverse effects of low light on tobacco seedlings via enhancing antioxidant and carbohydrate metabolism

**DOI:** 10.3389/fpls.2025.1666102

**Published:** 2025-09-02

**Authors:** Wenzheng Xu, Peng Li, Tuanwei Pu, Qiaozhen liu, Huige Han, Yanping Li, Zhaohui Wu

**Affiliations:** ^1^ Tobacco Research Institute, Henan Academy of Agricultural Sciences, Xuchang, China; ^2^ Nanyang Branch, Henan Provincial Tobacco Company, Nanyang, China; ^3^ Xuchang Branch, Henan Provincial Tobacco Company, Xuchang, China

**Keywords:** Nicotiana tabacum, low light, ROS, sugars, melatonin

## Abstract

There is relatively little knowledge about how melatonin helps tobacco withstand low light stress. To clarify this, a tobacco cultivar ZY100 was planted under light intensity of 150 μmol m^-2^ s^-1^ (low light) and 1000 μmol m^-2^ s^-1^ (control), and extra melatonin (200 μM) was applied to study the impacts of melatonin on tobacco seedlings under low light. Results showed that low light lowered plant height, stem thick, leaf number and shoot biomass, while melatonin alleviated these negative impacts of low light. Low light decreased net photosynthetic rate (*A*_N_), while melatonin application increased the *A*_N_ of low light-affected tobacco by reducing stomatal and non-stomatal limitations. Low light promoted *NtSOD*, *NtPOD* and *NtCAT* (encoding superoxide dismutase, catalase and peroxidase, respectively) expressions, and ascorbate (AsA) and glutathione (GSH) contents in tobacco leaves, which was beneficial for antioxidation in theory, however, higher O_2_^·-^ and H_2_O_2_ contents were still observed, damaging the *A*_N_. Melatonin application could further up-regulate *NtSOD*, *NtPOD* and *NtCAT* expressions and promote the AsA-GSH cycle by increasing ascorbate peroxidase and dehydroascorbate reductase activities in low light-affected tobacco leaves, lowering O_2_^·-^ and H_2_O_2_ contents. Because low light decreased the *A*_N_, lower leaf sucrose and starch contents were measured in low light-affected tobacco. And the decreased sucrose in low light-affected tobacco leaves was attributed to the down-regulated *NtSPS* (encoding sucrose phosphate synthase) expression, and the up-regulated *NtCWINV* (encoding cell wall invertase) expression. The reduced starch in low light-affected tobacco leaves was associated to the down-regulated *NtAGP* (encoding ADP-glucose pyrophosphorylase) and *NtGBSS* (encoding granule-bound starch synthase) expressions, and the up-regulated expression of *α-amylase.* Melatonin application could up-regulate *NtSPS* expression to promote sucrose synthesis and down-regulate *NtCWIN* expression to inhibit sucrose hydrolysis in low light-affected tobacco leaves, increasing leaf sucrose content. Moreover, melatonin application up-regulated *NtAGP* and *NtGBSS* expressions to enhance the starch biosynthesis, finally resulting in increased starch content in low light-affected tobacco leaves. These results indicated that melatonin application can alleviate the adverse effects of low light on tobacco growth via regulating antioxidant and carbohydrate metabolism.

## 1 Introduction

In order to optimize the use of limited land, intercropping has become one of the important agricultural planting patterns in China. And the intercropping system of wheat (T*riticum turgidum*), barley (*Hordeum vulgare*) and sweet potato (*Ipomoea batatas*), etc. with tobacco (*Nicotiana tabacum*) (food crop/tobacco) is the characteristic planting mode in the tobacco-growing areas of central China, which can not only guarantee grain production, but also produce characteristic cash crops (Liu et al., 2015). However, in the intercropping system, there is competition between different crops. Because the newly planted crops are at a lower spatial level, their competition for light is often weak (Abdel-Wahab and Abd El-Rahman, 2016), so the tobacco seedlings are often affected by low light during the growth of seedlings in the intercropping system.

As we all know, low light leads to the tobacco seedlings to develop weakly, resulting in significant changes in morphological traits. For example, Wu et al. (2021) reported that low light obviously inhibited the leaf number, dry weight, and leaf area, etc. And the negative influences of low light on morphological traits are strongly connected to the changes in intrinsic physiological metabolism. Among them, the most significant impact is that low light could cause obvious increases in the content of reactive oxygen species (ROS) in plants, especially in H_2_O_2_ and O_2_^·-^ levels (Liu et al., 2019; Raza et al., 2020), via reducing enzymes activities involved in antioxidant metabolism, such as catalase (CAT), peroxidase (POD) and superoxide dismutase (SOD), or via decreasing antioxidant substances contents, like ascorbate (AsA) and reduced glutathione (GSH) (Liu et al., 2019; Raza et al., 2020). And the reduced AsA or GSH level was linked to the limited activities of enzymes, such as ascorbate peroxidase (APX) and dehydroascorbate reductase (DHAR), etc. in the AsA-GSH cycle (Liu et al., 2019). In addition, the morphological formation of plants is closely associated with the supply capacity of photosynthetic products (Hu et al., 2024, 2025) and the light is a necessary factor for plant photosynthesis. Previous studies have reported that low light will reduce the photosynthetic efficiency of rapeseed (*Brassica compestris*) (Zhu et al., 2017), soybean (*Glycine max*) (Feng et al., 2019), wheat (Yang et al., 2020), and tobacco (Wu et al., 2021), etc. to limit plant growth. Hence, low light has been identified as an important factor that inhibits crop growth (Lu et al., 2019). Low light can alter the absorb of the light energy by reducing chlorophyll content (Feng et al., 2019), photosystem II (PSII) and photosystem I (PSI) complex contents (Wu et al., 2021), and limit CO_2_ fixation by restricting ribulose diphosphatecarboxylase (Rubisco) activity (Tang et al., 2022). Moreover, low light also inhibits the carbon metabolism by influencing the enzyme activities and gene expressions participating in the process of conversion from triose phosphate, the initial product of photosynthetic products, to other carbohydrates (Tang et al., 2022). For instance, low light decreased the activities of cytosolicfructose-1,6-bisphosphatase (FBPase), sucrose synthase (SuSy), and sucrose phosphate synthase (SPS), the major enzymes controlling the synthesis of sucrose, and the activities of ADP-glucose pyrophosphorylase (AGPase), starch-branching enzyme (SBE), soluble starch synthase (SSSase), and granule-bound starch synthase (GBSSase), the main enzymes regulating the synthesis of starch (Hendrix and Huber, 1986), finally decreasing sucrose and starch contents in leaves (Hendrix and Huber, 1986; Proietti et al., 2023).

Melatonin influences many physiological functions such as circadian sleep, food intake and immune system in animals (Lerner et al., 1958; Reiter et al., 2010). And it was reported in horticultural crops in 1995 (Dubbels et al., 1995) and has since been found in over 140 types of plants (Nawaz et al., 2016). Additionally, melatonin was found with obvious physiological and metabolic regulatory effects on crops, and the most important of which includes ROS clearance (Hu et al., 2021). Hence, the application of melatonin can alleviate the effects of abiotic stress such as cold (Bajwa et al., 2014), salt stress (Liang et al., 2015), high temperature (Shi et al., 2015b) and water deficit (Hu et al., 2022), on plants by reducing the accumulation of ROS through stimulating the enzyme system including SOD, CAT, and POD activities and nonenzymatic system such as the AsA-GSH cycle (Hu et al., 2021). Additionally, recent studies stated that extra melatonin application could also affect the carbohydrate metabolism of plants (Qian et al., 2015; Zhao et al., 2015; Li et al., 2018), and some researches noticed that exogenous melatonin application could enhance sugar metabolism in abiotic-stressed plants, thereby facilitating their growth (Shi et al., 2015a; Zhang et al., 2017). For example, Dawood and El-Awadi (2015) reported that exogenous melatonin promoted the leaf carbohydrate content of salt-stressed *Vicia faba* to enhance the growth of plants; Hu et al. (2016c) found that extra melatonin spraying regulated galactinol, mannobiose, and sorbose levels in *Cynodon dactylon* to promote its growth under cold conditions; and Hu et al. (2020) stated that exogenous melatonin promoted starch accumulation in male tissues of drought-stressed cotton (*Gossypium hirsutum*) to promote pollen fertility. Regarding low light stress, only a limited number of studies reported that extra melatonin spraying enhanced the resistance capacity of pepper (*Capsicum annuum*) (Li et al., 2022) and woodland strawberry (*Fragaria vesca*) (Shi et al., 2024) to low light by reducing ROS. There is no more information about exogenous melatonin affecting crops in response to low light conditions. Therefore, more research is needed to clarify the mechanism by which exogenous melatonin enhances the weak light resistance of crops.

In the present study, we hypothesized that extra melatonin supply would alleviate low light’s negative impacts on tobacco seedlings growth via rising ROS metabolic balance and carbohydrate balance. The objects of this study were intended to explore how exogenous melatonin influences the antioxidant (eg. antioxidant enzyme system and non-enzyme system related to ROS clearance) and carbohydrate metabolism (eg. sucrose metabolism and starch metabolism related to photosynthesis) in low light-affected tobacco seedlings. The expected results of this study will reveal the mechanism by which melatonin regulates plant growth and development under low light stress, and will fill the gap in its application on plants under low light stress.

## 2 Materials and methods

### 2.1 Treatments and sampling

An experiment was established in growth chambers using a tobacco cultivar ZY100 in Henan Academy of Agricultural Sciences. The growth chamber conditions were temperature at 25°C/20°C (day/night), air humidity at 75%, and light intensity at 1000 μmol m^-2^ s^-1^ (12 ^h^ per day). Seeds were sowed in a seedling tray. When seedlings had two true leaves, they were moved into 100 pots being filled with 10 kg clay soil, with one plant in each pot. After the transplanted seedlings have adapted for 7 days, 200 µM melatonin solution (since our previous pre-experiments indicated that this melatonin concentration could alleviate the effect of low light on tobacco seedlings) was applied randomly to seedlings in 50 pots under dark conditions in the evening, and other seedlings in remained 50 pots were sprayed with deionized water. The entire plant was sprayed, and each plant was sprayed with approximately 6–8 mL of melatonin or deionized water every two days. After spraying three times, each treatment was evenly and randomly divided into two groups. One of the groups was placed into the growth chamber under 1000 μmol m^-2^ s^-1^ (12 h per day) as conventional (control) light intensity, and another group was placed into a growth chamber having 150 μmol m^-2^ s^-1^ (12 h per day) as low light intensity (Demirevska et al., 2010). The other environment conditions for the two growth chambers are same as 25°C/20°C (day/night) and 75% air humidity. After 20 days, the morphological traits of seedlings were assayed. Moreover, the newest fully developed main stem leaves were used for the measurement of photosynthesis parameters. After the measurement of photosynthesis parameters, same leaves were collected for biochemical analysis and gene assay.

### 2.2 Determination of morphological traits

Plant height of tobacco defined as the distance from the rootstock to the top of the stem was determined using a meter stick. The vernier caliper was used to measure the thick of stem base. After dividing seedlings into aboveground and underground parts, the seedlings were heated at 105 °C for 30 min, and then for 48 h at 75 °C. The weight of dry samples was measured by a balance.

### 2.3 Measurement of photosynthesis

Net photosynthetic rate (*A*_N_), stomatal conductance (*G*s) and intercellular CO_2_ concentration (*C*i) were detected by a Li-6400 photosynthesis equipment (Li-COR, USA) with leaf chamber conditions: 25°C leaf temperature, 1000 μmol m^-2^ s^-1^ light intensity, air flow rate at 500 μmol s^-1^, 75% relative humidity of air and 400 µmol mol^-1^ reference CO_2_ concentration when the measurement system reached steady-state conditions.

### 2.4 Assay of ROS and malondialdehyde contents

The assay of leaf O_2_^·-^ level was conducted as previously described (Zhang et al., 2023). Briefly, leaves (0.2-0.3 g, fresh weight) were crushed with liquid nitrogen into powder, before being extracted with phosphate buffer solution (3 mL, 50 mM) with a pH of 7.8. After performing a centrifugation at 4 °C for 15 min at 10,000 g, the liquid layer was used for determining O_2_^·-^ content based on the technique of hydroxylamine oxidation.

The measurement of H_2_O_2_ content was conducted as previously described (Zhang et al., 2010). Briefly, fresh leaf samples (0.2-0.3 g) were ground with 1.5 mL acetone before a centrifugation (10,000 *g* for 15 min). Then, 1 mL supernatant was mixed with 0.1 mL Ti_2_SO_4_ (5%) and 0.2 mL NH_4_OH before a centrifugation was conducted at 10–000 g for 10 min. The precipitates were washed with acetone until colorless before the precipitates were dissolved by 2 N H_2_SO_4_. After measuring the absorbance at A_415_, the H_2_O_2_ content could be calculated.

The MDA assay was referred to Hu et al. (2016b). Briefly, fresh leaves (0.2-0.3 g) were crushed with 2 mL trichloroacetic acid (8%) into a homogenate before performing a 10,000 g centrifugation for 12 min. Subsequently, 2 mL supernatant was boiled for 15 min with 7 mL thiobarbituric acid (0.6%) before a 10,000 g centrifugation was performed at 4 °C for 12 min. After being cooled, the absorbance was detected at 600, 532 and 450 nm, respectively, for the calculation of MDA content as 6.45*(OD532-OD600)-0.56*OD450.

### 2.5 Determination of carbohydrates

Leaf carbohydrates were extracted referring to Hu et al. (2018). Briefly, 1 mL ethanol (80%, v/v) and dry leaf powder (40–45 mg) were incubated for three times at 80 °C. Subsequently, the supernatants from the three extractions were merged. Then, 80% ethanol was used to calibrate the extraction to 3 mL. After adding 30 mg activated charcoal to absorb impurities such as chlorophyll that may affect the final absorbance, a 1164 g centrifugation was performed for 15 min. Then, 20 μL extract was pipetted into a microplate. After an incubation at 45 °C, distilled water (20 μL) was pipetted into each cell in the microplate. For the assessment of glucose, fructose and sucrose, the mixtures were incubated three times for 15, 15 and 60 min, respectively, at 30 °C. In addition, glucose assay reagent (100 μL), phosphoglucose isomerase (10 μL, 0.25 U), and invertase (10 μL, 83 U) were added respectively before each heating. After each heating, the absorbance at 340 nm was detected.

The above residues insoluble in alcohol were collected for the determination of starch. The residues were boiled with 1 M KOH (0.5 mL) for 1 h before regulating pH to 6.5-7.5. Immediately after that, 100 μL α-amylase was pipetted to the mixture before a centrifugation was performed for 60 min at 65 °C. Subsequently, the acetic acid was utilized to regulate the pH less than 5 before adding amyloglucosidase (0.25 mL) and centrifugating at 55 °C for 60 min. Then, a 10,000 g centrifugation was performed for 15 min. The upper layer solution was collected for detecting glucose concentration. The starch content could be calculated based the glucose concentration (Hu et al., 2018).

### 2.6 Assay of APX and DHAR activities

The crude enzyme solution of APX and DHAR were obtained as previously described (Djanaguiraman et al., 2009). Then, APX activity was detected via assaying the reaction amount of AsA in 3 mL reaction solution containing 200 µL enzyme extract, 2.5 mM H_2_O_2_, 0.1 mM sodium ascorbate, 50 mM sodium phosphate (pH 7.0), and 0.1 mM EDTA at A_290_ (Hu et al., 2016b).

The reaction mixture for DHAR activity contained 0.05 mL enzyme extract, 0.05 mL reduced glutathione (50 mM), 0.05 mL DHA (4 mM), and 0.85 mL potassium phosphate (100 mM, pH 7.8). The DHAR activity was assayed by detecting the DHA reduction at A_265_ (Hu et al., 2016b).

### 2.7 Relative expression of genes

Leaf RNA was extracted by A Plant Total RNA lsolation Kit from the Vazyme Company (Nanjing, China). The generation of cDNAs was completed with a cDNA Synthesis Kit from the Vazyme Company. The quantitative RT-PCR was conducted using a fluorescence quantitative kit Green™ Premix Ex Taq™ II from the Vazyme Company according to Yu et al. (2024). The expression of *NtSOD*, *NtPOD*, *NtCAT*, *NtSPS*, *NtSuSy*, *NtCWINV*, *NtADP*, *NtSSS*, *NtGBSS*, *β-amylase* and *α-amylase* encoding SOD, POD, CAT, SPS, SuSy, cell wall invertase, AGPase, SSSase, GBSSase, *β*-amylase and *α*-amylase, respectively, were detected. The gene *Nttubulin* was selected as the housekeeping gene. **Table 1** showed the used primers for our study. The gene relative expression was obtained through the use of the method of 2^-△△Ct^ (Livak and Schmittgen, 2001).

**Table 1:** Gene primer sequences used for the quantitative real-time PCR analysis.

Gene	Forward primer (5′-3′)	Reverse primer (5′-3′)
*NtSOD*	GACGGACCTTAGCAACAGG	CTGTAAGTAGTATGCATGTTC
*NtPOD*	CTCCATTTCCATGACTGCTTTG	GTTGGGTGGTGAGGTCTTT
*NtCAT*	CACCTTACCTGTGCTGATTTC	CTGGTGTAGAACTTGACAGC
*NtSPS*	ATCTTGAAAGGGGCTGTCGA	CGTTTCCGCTGGTATACGTG
*NtSuSy*	CTCAACATCACCCCTCGAAT	ACCAGGGGAAACAATGTTGA
*NtCWINV*	CTTACACCCAATTACCGGCG	GACACTCTTTTGGGTCGTCG
*NtADP*	AGCAAAGACGTGATGTTAAACC	TCTTCACATTGTCCCCTATACG
*NtSSS*	TGAGTTCAGGTGGTCTTGTCTTTGG	AATAGCCCTTATGCGTCGATGATGG
*NtGBSS*	AACAGCTCGAAGTGTTGTA	ATCTGCTTGGAACCAACATAA
*α-amylase*	ATATTGCAGGCCTTCAACTGGG	TGGAAGGTAACCTTCAGGAGACAA
*β-amylase*	TGAGCTATTGGAAATGGCGAAGA	AAGAGGGATCGTGCAGGAATCA
*Nttubulin*	GCATCTTTGCGTACACTTTGCT	ACATAAGCCCAAAACTAGCTGGA

### 2.8 Data analyses

The SPSS statistic software (Version 17.0, SPSS Inc., USA) was used to conduct the one-way analysis of variance with least significant difference (LSD) test (*P*<0.05). Graphs were made by the software Origin 8.0 (Origin Lab Inc., USA).

## 3 Results

### 3.1 Influences of melatonin on agronomic traits of low light-affected tobacco seedlings

Under control light intensity, the melatonin spraying increased shoot biomass and root biomass (**Table 2**). Compared with control light intensity, low light significantly decreased the height of plant, stem thick, leaf number and shoot biomass while did not alter root biomass (**Table 2**). Extra melatonin alleviated the reduction in plant height, leaf number and shoot biomass caused by low light, because plant height, leaf number and shoot biomass of low light-affected tobacco seedlings increased by 65.4%, 35.7% and 55.6%, respectively, after the melatonin application (**Table 2**).

**Table 2:** Effects of melatonin on agronomic traits of low light-stressed tobacco seedlings.

Light intensity	Melatonin (µM)	Plant height (cm)	Stem thick (mm)	Leaf number (no.)	Shoot biomass (g)	Root biomass (g)
CK	0	18.17 ± 0.60a	2.87 ± 0.07a	7.33 ± 0.33a	2.03 ± 0.09b	0.12 ± 0.03b
	200	19.03 ± 0.99a	3.03 ± 0.12a	7.67 ± 0.33a	2.33 ± 0.09a	0.19 ± 0.02a
Low light	0	8.67 ± 0.67c	2.13 ± 0.09b	4.67 ± 0.33c	0.60 ± 0.06d	0.07 ± 0.01b
	200	14.33 ± 0.88b	2.46 ± 0.09b	6.33 ± 0.33b	0.93 ± 0.03c	0.10 ± 0.01b

Different lower-case letters within the same column represent significant differences at the *P* < 0.05 level. Values are means ± standard error (SE, n = 3).

### 3.2 Influences of melatonin on the photosynthesis of low light-affected tobacco seedlings

**Figure 1:**
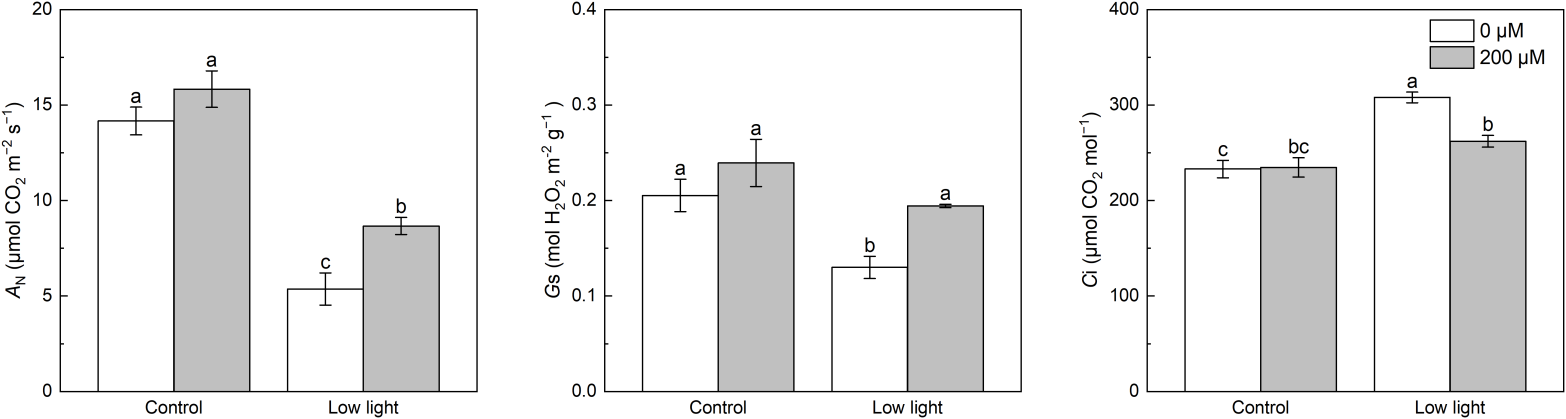
Effect of melatonin application on gas exchange parameters including net photosynthetic rate (*A*_N_), stomatal conductance (*g*_s_), and intercellular CO_2_ concentration (*C*_i_) of tobacco seedlings under low light. Different letters represent significant differences at the *P* < 0.05 level. Values are means ± SE (n = 3).

Under control light intensity, extra melatonin application did not alter the *A*_N_, *G*s and *C*i (**Figure 1**). Compared with control light intensity, *A*_N_ and *G*s were obviously decreased while *C*i was obviously increased by low light. Exogenous application of melatonin increased *A*_N_ and *G*s in low light-affected tobacco seedlings by 61.5% and 49.2%, respectively. However, exogenous application of melatonin decreased the *C*i in low light-affected tobacco seedlings by 14.9%.

### 3.3 Influences of melatonin on ROS and MDA contents of low light-affected tobacco seedlings

**Figure 2:**
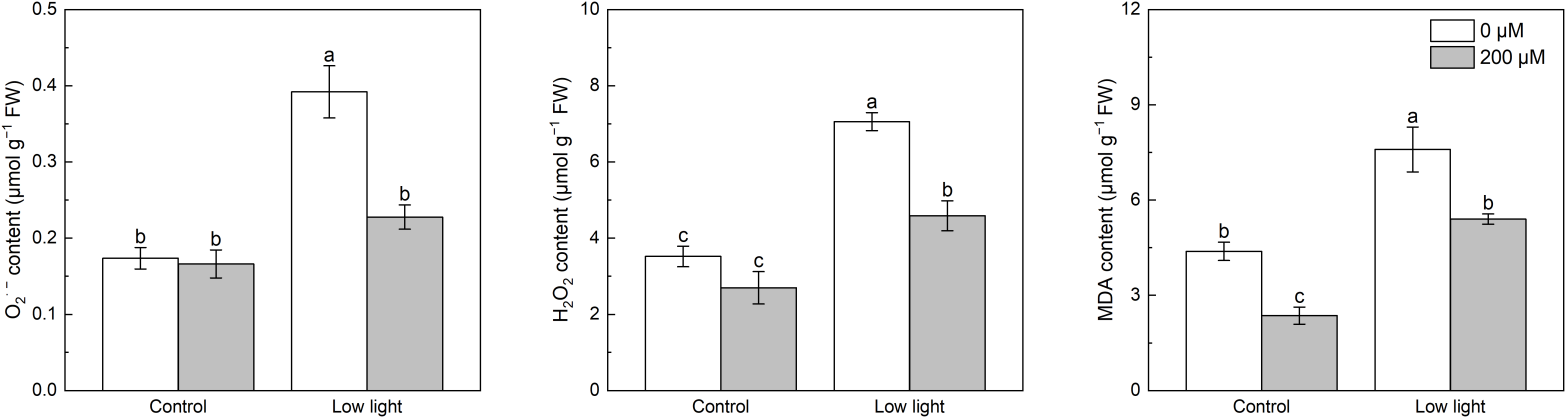
Effect of melatonin application on the content of leaf superoxide anion (O_2_^·-^), hydrogen peroxide (H_2_O_2_) and malonaldehyde (MDA) in tobacco seedlings under low light. Different letters represent significant differences at the *P* < 0.05 level. Values are means ± SE (n = 3).

Under control light intensity, extra melatonin application did not alter the O_2_^·-^ and H_2_O_2_ levels (**Figure 2**). Compared with control light intensity, low light significantly increased O_2_^·-^ and H_2_O_2_ levels by 136.0% and 161.6%, respectively. Spraying additional melatonin lowered the O_2_^·-^ and H_2_O_2_ levels in low light-affected tobacco seedlings by 41.9% and 35.0%, respectively.

Under control light intensity, the melatonin spraying lowered the MDA content by 46.2% (**Figure 2**). The MDA content of low light-treated seedlings increased by 221.8% as compared with seedlings under control light intensity, but melatonin application prevented the increase in MDA content caused by low light.

### 3.4 Influences of melatonin on antioxidant system of low light-affected tobacco seedlings

**Figure 3:**
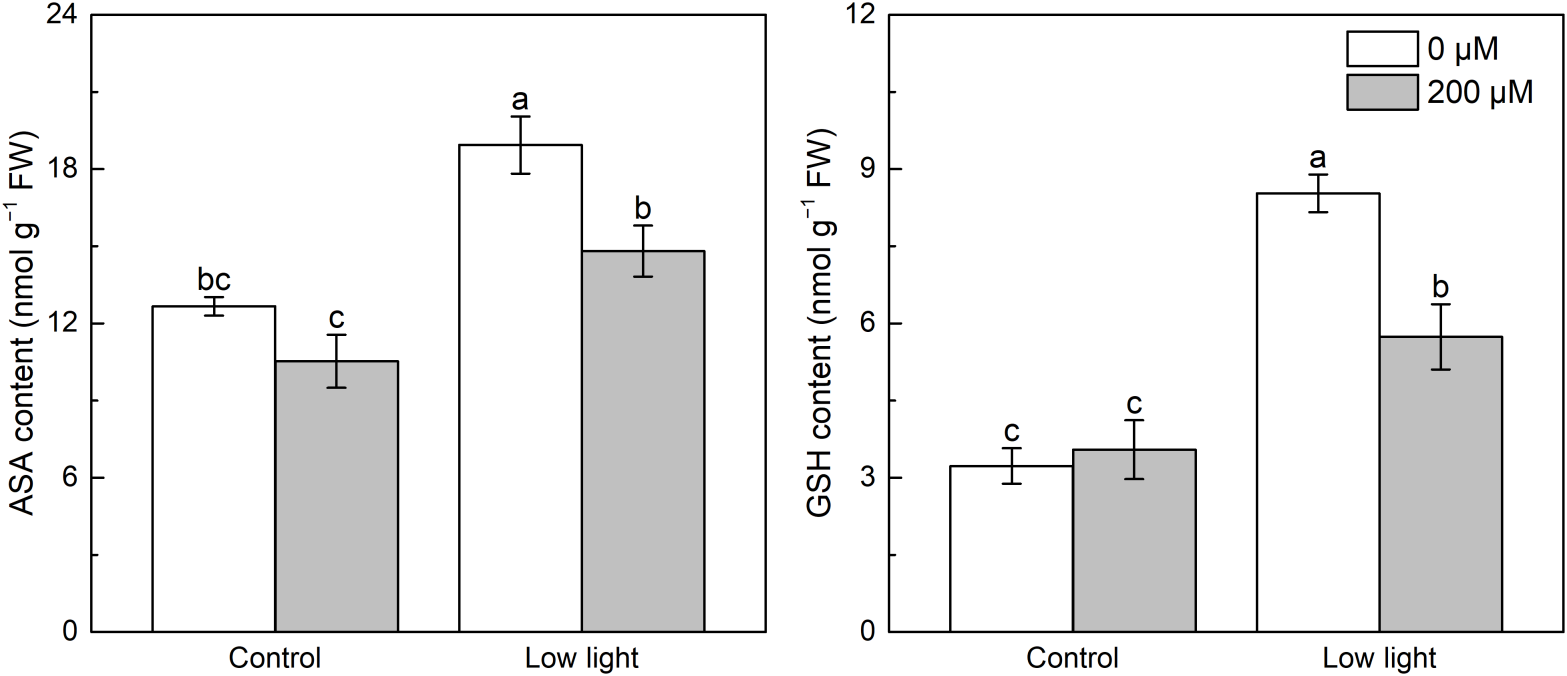
Effect of melatonin application on the content of leaf ascorbate (AsA) and glutathione (GSH) in tobacco seedlings under low light. Different letters represent significant differences at the *P* < 0.05 level. Values are means ± SE (n = 3).

Under control light intensity, extra melatonin application did not alter the ASA and GSH contents (**Figure 3**). Compared with control light intensity, leaf ASA content was 79.8% higher for the low light intensity conditions. Application of melatonin significantly decreased ASA accumulation in low light-affected seedlings. In addition, a substantial increase with 140.6% in GSH content was detected in low light-affected tobacco seedlings compared with those seedlings under control light intensity, however, the addition of melatonin alleviated this effect.

**Figure 4:**
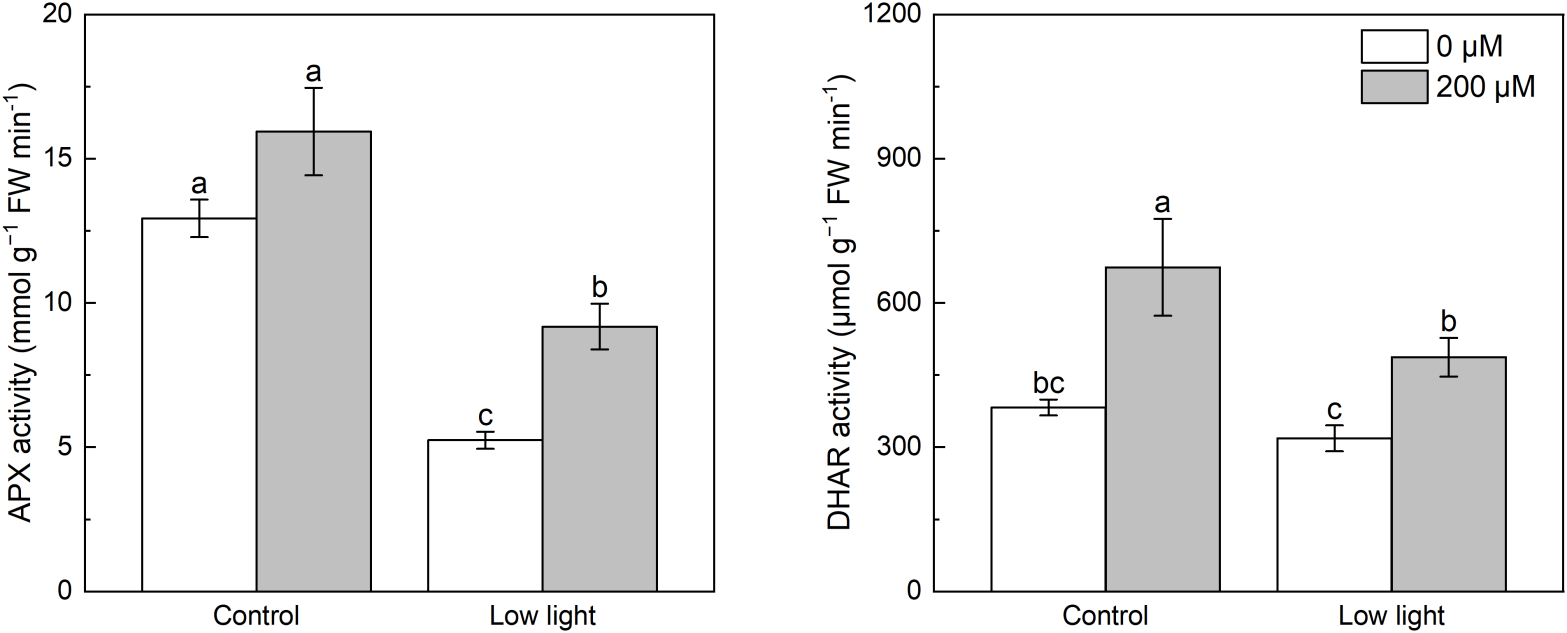
Effect of melatonin application on the activity of ascorbate peroxidase (APX) and dehydroascorbate reductase (DHAR) in tobacco seedlings under low light. Different letters represent significant differences at the *P* < 0.05 level. Values are means ± SE (n = 3).

Under control light intensity, melatonin application did not alter the APX activity (**Figure 4**). However, the APX activity was obviously reduced by low light. Application of melatonin markedly increased the APX activity in low light-affected seedlings. Low light had no influence on the DHAR activity in tobacco seedlings compared with control light intensity (**Figure 4**). Melatonin spraying promoted the DHAR activity in seedlings under control light intensity and low light.

**Figure 5:**
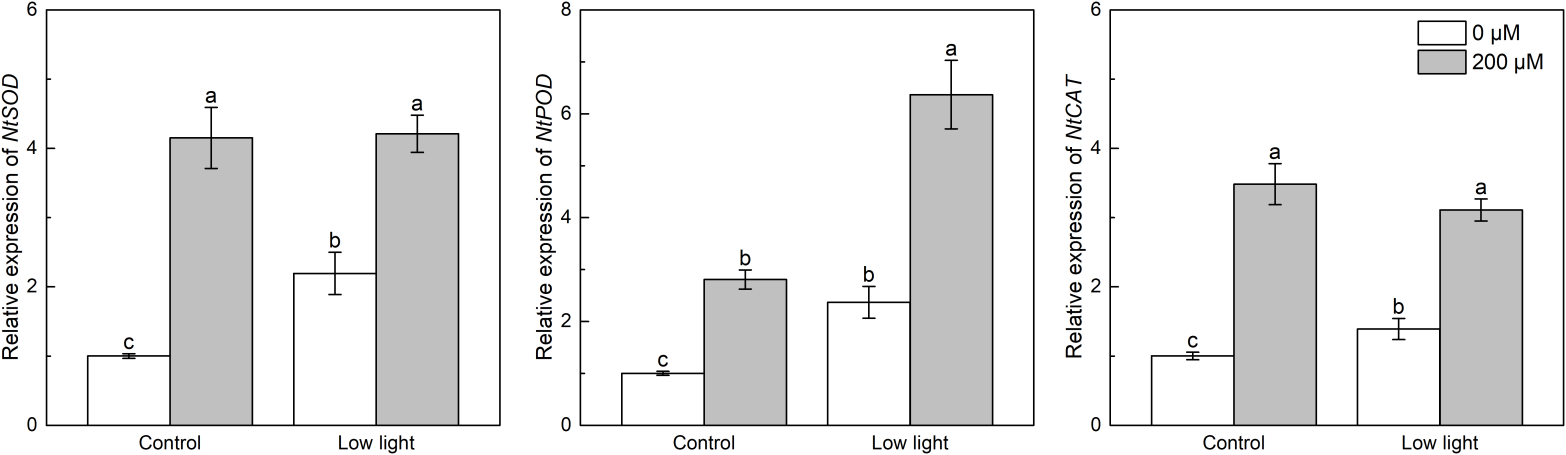
Effect of melatonin application on the expression of genes *NtSOD*, *NtPOD* and *NtCAT* encoding superoxide dismutase peroxidase and catalase, respectively, in tobacco seedlings under low light. Different letters represent significant differences at the *P* < 0.05 level. Values are means ± SE (n = 3).

Under control light intensity, melatonin spraying significantly increased the expression level of *NtSOD*, *NtPOD* and *NtCAT* (**Figure 5**). *NtSOD*, *NtPOD* and *NtCAT* expressions were significantly higher in low light-affected seedlings compared with those seedlings under control light intensity (**Figure 5**). Moreover, *NtSOD*, *NtPOD* and *NtCAT* expressions were promoted by the addition of melatonin in the low light-affected tobacco seedlings.

### 3.5 Influences of melatonin on leaf carbohydrate metabolism of low light-affected tobacco seedlings

**Figure 6:**
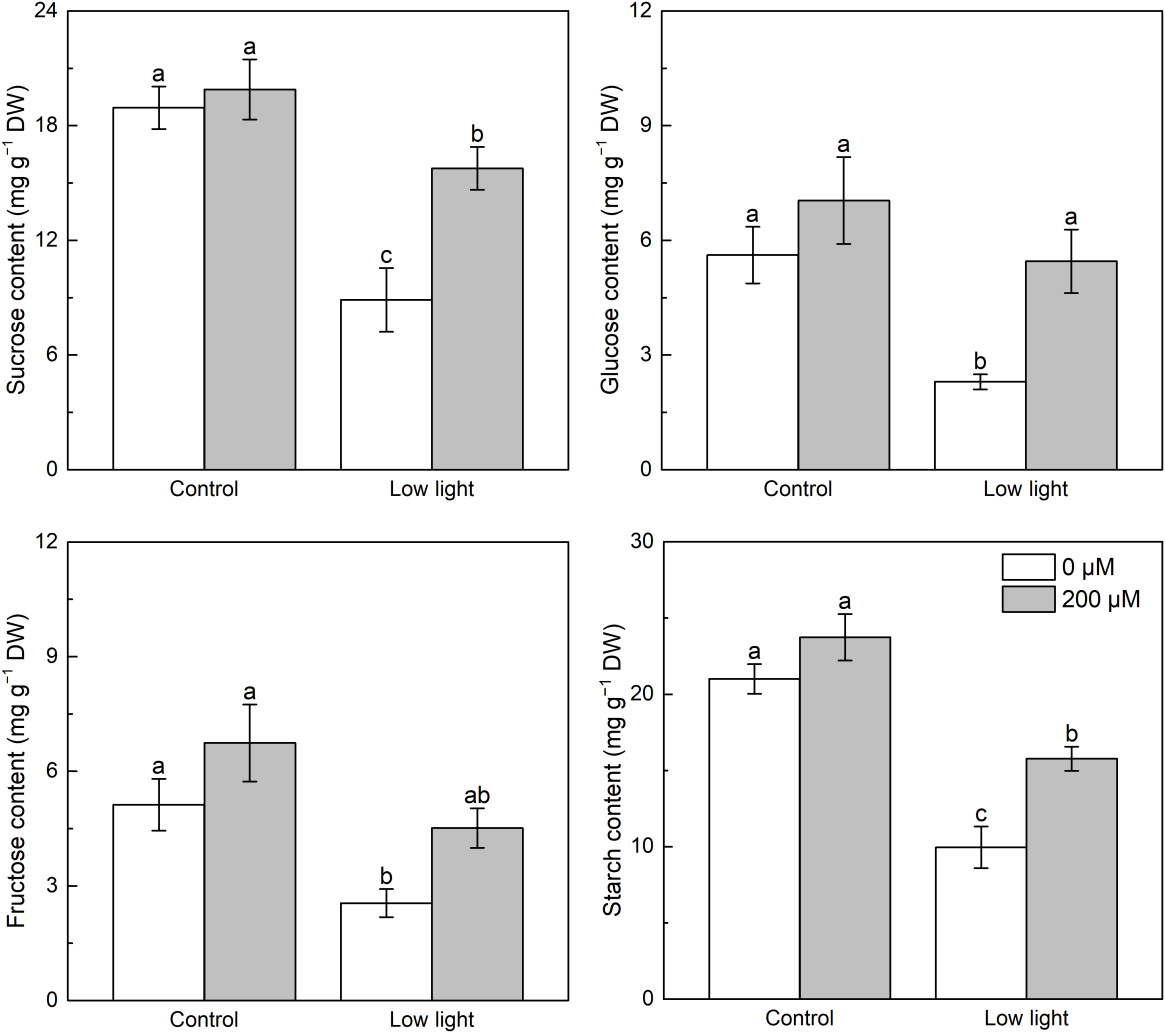
Effect of melatonin application on the content of leaf glucose, fructose, sucrose and starch in tobacco seedlings under low light. Different letters represent significant differences at the *P* < 0.05 level. Values are means ± SE (n = 3).

Under control light intensity, melatonin application had no influence on sucrose, fructose, glucose as well as starch contents (**Figure 6**). The level of sucrose, glucose, fructose and starch was markedly reduced by 55.3%, 67.4%, 62.2%, and 58.1%, respectively, by low light in comparison with the control light intensity. Although melatonin spraying had no influence on fructose content under low light, it increased sucrose, glucose, and starch contents of low light-affected seedlings.

**Figure 7:**
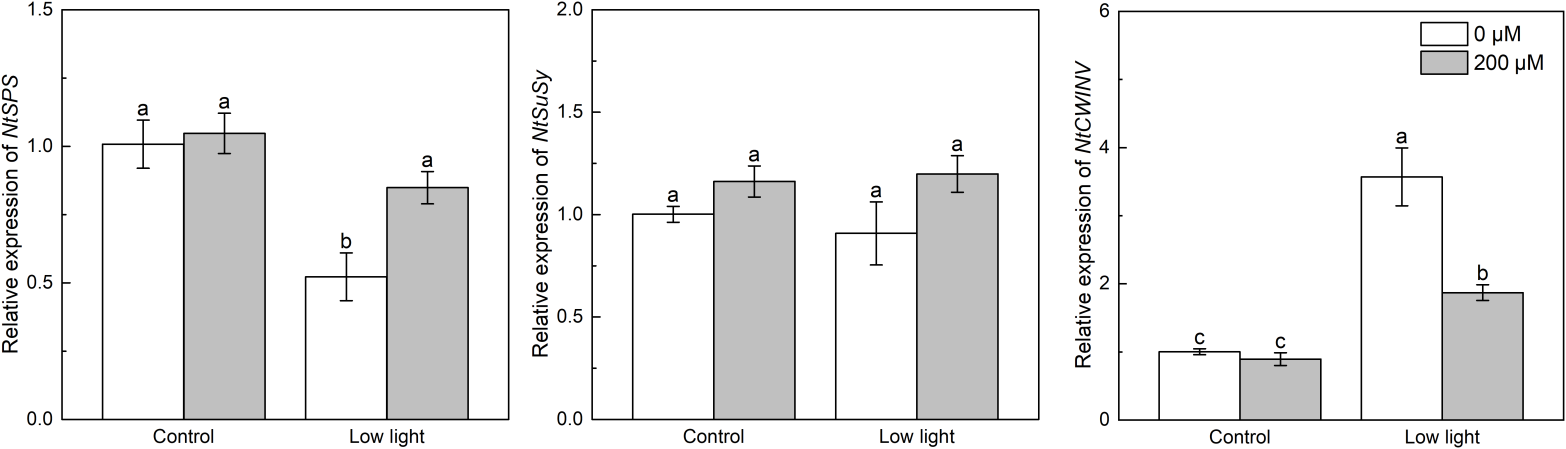
Effect of melatonin application on the expression of genes *NtSPS, NtSuSy* and *NtCWINV* encoding sucrose phosphate synthase, sucrose synthase, and cell wall invertase, respectively, in tobacco seedlings under low light. Different letters represent significant differences at the *P* < 0.05 level. Values are means ± SE (n = 3).

Under control light intensity, the expression of *NtSPS*, *NtSuSy* and *NtCWINV* was not influenced by extra melatonin (**Figure 7**). The expression of *NtSPS* was reduced by low light, while the *NtCWIN* expression was promoted by low light. And the *NtSuSy* expression was not impacted by low light. The expression of *NtSPS* in low light-affected seedlings was up-regulated by extra melatonin, but the expression of *NtCWIN* in low light-affected seedlings was down-regulated by melatonin application.

**Figure 8:**
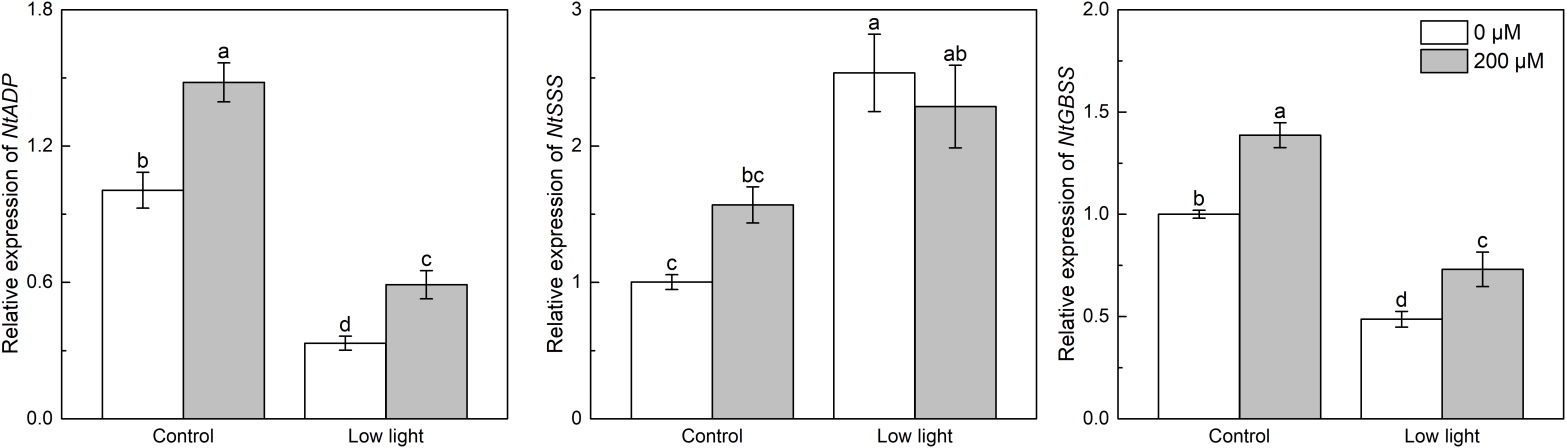
Effect of melatonin application on the expression of genes *NtADP*, *NtSSS* and *NtGBSS* encoding ADP-glucose pyrophosphorylase, soluble starch synthase and granule-bound starch synthase, respectively, in tobacco seedlings under low light. Different letters represent significant differences at the *P* < 0.05 level. Values are means ± SE (n = 3).

**Figure 9:**
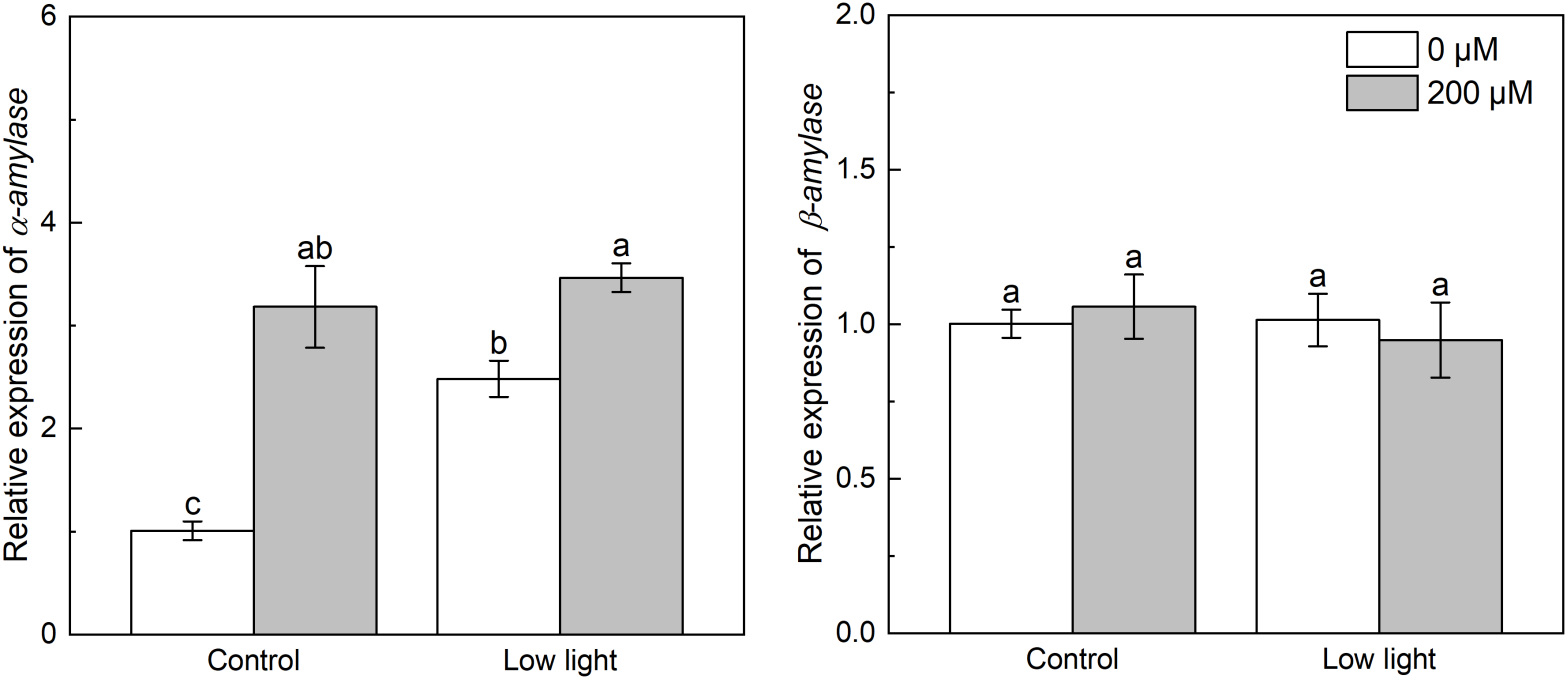
Effect of melatonin application on the expression of genes *α-amylase* and *β-amylase* encoding *α*- and *β*-amylase, respectively, in tobacco seedlings under low light. Different letters represent significant differences at the *P* < 0.05 level. Values are means ± SE (n = 3).

Under control light intensity, the expression of *NtADP* and *NtGBSS* was decreased by extra melatonin (**Figure 8**). The expression of *NtADP* and *NtGBSS* was down-regulated by 77.5% and 64.8%, respectively, by low light while the *NtSSS* expression was increased by 61.7% by low light. The *NtADP* and *NtGBSS* expressions in low light-affected seedlings were promoted by 77.2% and 50.0%, respectively, by exogenous melatonin. The expression of *α-amylase* was markedly increased by low light (**Figure 9**). Exogenous melatonin up-regulated the expression of *α-amylase* by 215.5% and 39.5% under control light intensity and low light, respectively. The *β-amylase* expression was not impacted by low light or exogenous melatonin (**Figure 9**).

## 4 Discussion

Previous studies have reported that light intensity will influence plants morphologically, and plant height, leaf number and plant biomass are usually closely related to light intensity (Zervoudakis et al., 2012; Ding et al., 2013). In support of above studies, results in the present study showed that low light notably reduced plant height, the thick of stem, the number of leaf and shoot biomass in relation to control light intensity (**Table 2**), indicating that low light inhibited the growth of tobacco seedlings (Wu et al., 2021). Many experiments have found that extra melatonin supply can help plants to resist abiotic stresses (Shi et al., 2015a; Zhang et al., 2017), and Li et al. (2022) stated that melatonin supply enhanced the growth of pepper seedlings under low light, which was manifested as higher aboveground and underground biomass. In the current study, although the root biomass of low light-affected seedlings was not affected by melatonin application, the plant height, leaf number and shoot biomass of low light-affected seedlings were increased by extra melatonin with 65.4%, 35.7% and 55.6%, respectively (**Table 2**), which suggested that extra melatonin primarily weakened the influences of low light on the growth of above-ground part, but not the underground part, for tobacco seedlings.

Photosynthesis is the fundamental physiological process for crop growth, and the products of photosynthesis are the main material source for the accumulation of plant biomass (Hu et al., 2024). Many studies found that crop photosynthetic capacity reduced markedly when exposed to low light (Ding et al., 2013; Feng et al., 2019). In our study, leaf *A*_N_ was also significantly inhibited by low light (**Figure 1**), which could explain the restricted growth of tobacco seedlings. Previous studies have shown that low light will limit photosynthesis by stomatal factors and non-stomatal factors (Ding et al., 2013). Hu et al. (2016a) reported that when the decreased *A*_N_ is accompanied by decreased *G*s as well as *C*i, the reduction in *A*_N_ is mostly caused by stomatal limitation; when the decreased *A*_N_ together with a decreased *G*s and an increased Ci was observed, non-stomatal limitation plays the dominant role in the reduction of *A*_N_. In this study, leaf *A*_N_ and *g*_s_ were significantly reduced by low light (**Figure 1**), while leaf *C*i was increased by low light, implying that the reduction in *A*_N_ of tobacco leaves under low light was mainly caused by non-stomatal factors in this study. Shi et al. (2024) claimed that melatonin spraying will not alter the leaf *A*_N_ of strawberry under normal light while significantly promotes the leaf *A*_N_ of strawberry under low light. Similarly, our study found that under control light intensity, extra melatonin did not influence the *A*_N_ (**Figure 1**), but increased the *A*_N_ in low light-affected tobacco seedlings by 61.5%, finally resulting in increased plant height, leaf number and shoot biomass in low light-affected seedlings. In addition, under low light, *g*_s_ was enhanced by melatonin application, while the *C*_i_ was significantly reduced by melatonin application (**Figure 1**), meaning that under low light, the enhancement of melatonin on *A*_N_ was not only attributed to the decreased stomatal limitation, but also to the reduction of non-stomatal limitation. This supported the previous study of Shi et al. (2024) where extra melatonin could mitigate the harmful effects of low light on *A*_N_ via decreasing stomatal and non-stomatal limitations.

Excessive ROS in leaves will damage the mechanism that conducts photosynthesis, which is a key factor leading to the reduction of photosynthesis (Hu et al., 2024; Yang et al., 2024). Past studies have confirmed that low light could lead to ROS accumulation, thereby causing oxidative stress to lower photosynthesis (Yang et al., 2008; Ding et al., 2013). Similarly, our results indicated that low light led to higher leaf O_2_^·-^ and H_2_O_2_ contents compared with control light intensity, resulting in membrane lipid peroxidation, so the increased MDA content was observed (**Figure 2**). In order to eliminate ROS, higher plants evolves efficient antioxidant system including enzymatic system and non-enzymatic system (Zhang et al., 2023). In the antioxidant enzyme system, SOD, POD and CAT have been found to play key roles. SOD mainly catalyzes the reduction of O_2_^·-^ to yield H_2_O_2_ and O_2_, and CAT and POD can exclusively scavenge H_2_O_2_ to form O_2_ (Gill and Tuteja, 2010). In the non-enzymatic system, AsA can effectively scavenge H_2_O_2_ (Li et al., 2010) and GSH can be further converted into AsA under the catalysis of enzyme DHAR (Foyer and Noctor, 2005). Previous studies have reported that low light increased SOD and POD activities in *Cucumis sativus* leaves (Ding et al., 2013), POD and CAT activities and ASA content in dragon spruce (*Picea asperata*) leaves (Yang et al., 2008), and GSH content in wheat leaves (Toldi et al., 2019). The findings of the current study correspond to above studies, since low light up-regulated *NtSOD*, *NtPOD* and *NtCAT* expressions, and promoted AsA and GSH contents in tobacco leaves than control light intensity, which would theoretically accelerate the clearance of O_2_^·-^ and H_2_O_2_. However, leaf O_2_^·-^ and H_2_O_2_ contents were still higher under low light than control light intensity, which might be because the increased ROS clearance rate caused by the enhanced antioxidant system was lower than the increased ROS generation rate caused by low light (Yang et al., 2008; Ding et al., 2013). Some studies showed that extra melatonin application can activate SOD, POD and CAT activities in strawberry (Shi et al., 2024) and promote AsA content in pepper (Li et al., 2022) to reduce O_2_^·-^ and H_2_O_2_ contents in low light-affected plants. In support of above reports, results of the current study presented that extra melatonin spraying further up-regulated *NtSOD*, *NtCAT* and *NtPOD* expressions in low light-affected tobacco leaves (**Figure 5**), finally leading to less O_2_^·-^ and H_2_O_2_ accumulation. Moreover, extra melatonin application decreased the content of AsA in low light-affected tobacco leaves (**Figure 3**), which should be because that extra melatonin application enhanced the activity of APX in low light-affected tobacco leaves (**Figure 4**), thereby promoting the reaction between AsA and H_2_O_2_, resulting in lower accumulation of H_2_O_2_ and AsA. In addition, extra melatonin application promoted the activity of DHAR in low light-affected tobacco leaves, meaning that extra melatonin application enhanced the conversion of GSH into AsA, thereby reducing the content of GSH.

Sucrose is an important product of photosynthesis. Low light caused lower leaf *A*_N_ in relation to control light intensity (**Figure 1**), so lower sucrose content in leaves was measured under low light compared with control light intensity. The biosynthesis of sucrose is catalyzed by SPS and SuSy (Zhang et al., 2024). Past studies found that low light decreased the activity of SPS and sucrose synthetase (SuSy) (Feng et al., 2019; Yang et al., 2025). Results in this study partially supported the previous reports, as low light did not affect the expression of *NtSuSy*, but lowered the expression of *NtSPS* (**Figure 7**), which would inhibited sucrose synthesis. Moreover, the hydrolysis of sucrose into glucose and fructose is regulated by cell wall invertase (CWINV) (Zhang et al., 2024). Results here indicated that low light up-regulated the expression of *NtCWINV*, accelerating the hydrolysis of sucrose. Hence, the combined effect of restricted sucrose synthesis and accelerated sucrose hydrolysis brought the lower sucrose content in low light-affected tobacco leaves. Surprisingly, lower leaf glucose and fructose levels were found in low light-affected tobacco, which should be because a large amount of glucose and fructose content will be used for respiration to resist abiotic stress (Zhang et al., 2024). Extra melatonin up-regulated the expression of *NtSPS*, decreased the *NtCWINV* expression, and had no marked impacts on the *NtSuSy* expression in low light-affected tobacco leaves, meaning that melatonin application promoted sucrose synthesis and inhibited sucrose hydrolysis in low light-affected tobacco leaves, so increased sucrose content was measured in melatonin-treated tobacco leaves under low light.

Starch is another main product of photosynthesis apart from sucrose. The biosynthesis of starch was mainly regulated by three enzymes including AGPase catalyzing the glucose-1-phosphate to yield ADP-glucose, and SSSase and GBSSase catalyzing the generation of amylose and amylopectin from the ADP-glucose (Hu et al., 2020), and the hydrolysis of starch into hexose was mainly regulated by α-amylase and β-amylase (Hammond and Burton, 1983). Low light increased the expression of *NtSSS*, implying that low light could enhance the generation of amylopectin from ADP-glucose. However, low light down-regulated the expression of *NtAGP* (**Figure 8**), meaning that the production of ADP-glucose was restricted, consequently inhibiting starch biosynthesis. Moreover, the expression of *NtGBSS* was restricted, which could further restrict the amylose biosynthesis. Regarding starch degradation, although low light did not influence *β-amylase* expression, but up-regulated *α-amylase* expression (**Figure 9**), which could accelerate the hydrolysis of starch. These could explain the lower leaf starch content in low light-affected tobacco than control ones. Similarly, previous studies reported that low light resulted in low leaf starch content in soybean (Feng et al., 2019) and wheat (Yang et al., 2023). Melatonin application could obviously up-regulated *NtAGP* and *NtGBSS* expressions in low light-affected tobacco leaves, which could promote the starch biosynthesis. Hence, melatonin application increased the content of starch in low light-affected leaves. Moreover, despite the *β-amylase* expression in low light-affected tobacco leaves was not influenced by extra melatonin, the *α-amylase* expression in low light-affected tobacco leaves was further enhanced by melatonin application, which could promote the decomposition of starch into glucose, explaining the higher glucose level in low light-affected tobacco leaves with melatonin application than without melatonin application.

## 5 Conclusion

Low light led to lower plant height, stem thick, leaf number and shoot biomass, while melatonin application promoted plant height, leaf number and shoot biomass of low light-affected tobacco seedlings. Leaf *A*_N_ was decreased by low light, while the *A*_N_ of low light-affected tobacco was increased by melatonin application via reducing both stomatal limitation and non-stomatal limitation. Low light resulted in higher O_2_^·-^ and H_2_O_2_ contents in tobacco leaves, damaging the *A*_N_. Melatonin application up-regulated the expression of *NtSOD*, *NtPOD* and *NtCAT* and promoted APX and DHAR activities in low light-affected tobacco leave to lower O_2_^·-^ and H_2_O_2_ contents. Since low light decreased the leaf *A*_N_, lower leaf sucrose and starch contents were measured under low light. And the lower sucrose in low light-affected tobacco leaves was attributed to the inhibited sucrose synthesis caused by down-regulated *NtSPS*, and the accelerated sucrose hydrolysis caused by up-regulated *NtCWINV*. The lower starch in low light-affected tobacco leaves was related to the inhibited starch synthesis caused by down-regulated *NtAGP* and *NtGBSS* expressions, and the accelerated starch hydrolysis caused by up-regulated *α-amylase.* Melatonin application could up-regulate *NtSPS* expression to promote sucrose synthesis and down-regulate *NtCWIN* to inhibit sucrose hydrolysis in low light-affected tobacco leaves, enhancing leaf sucrose content of tobacco under low light. Melatonin application up-regulated *NtAGP* and *NtGBSS* expressions to enhance the starch biosynthesis, finally increasing leaf starch content for low light-affected tobacco seedlings. Therefore, our study found that exogenous melatonin can alleviate harmful impacts of low light on tobacco seedlings by regulating antioxidant metabolism and carbohydrate metabolism. Of course, the effect of melatonin on the carbohydrate metabolism of cotton seedlings under low light conditions may also affect the energy metabolism of the seedlings to influence the growth of tobacco seedlings, because carbohydrates are the material basis for energy metabolism. This can be further explored in future research.

## Data Availability

The original contributions presented in the study are included in the article/Supplementary Material. Further inquiries can be directed to the corresponding author.
